# An Accurate Book Spine Detection Network Based on Improved Oriented R-CNN

**DOI:** 10.3390/s24247996

**Published:** 2024-12-14

**Authors:** Haibo Ma, Chaobo Wang, Ang Li, Aide Xu, Dong Han

**Affiliations:** 1 Library, Panjin Campus of Dalian University of Technology, Panjin 124000, China; mahaibo@dlut.edu.cn (H.M.); handong21@dlut.edu.cn (D.H.); 2 School of Information Science and Technology, Dalian Maritime University, Dalian 116026, China; cbwang@dlmu.edu.cn (C.W.); liangahvl@dlmu.edu.cn (A.L.)

**Keywords:** book spine detection, oriented R-CNN, deformable convolution, secondary feature fusion, K-median clustering

## Abstract

Book localization is crucial for the development of intelligent book inventory systems, where the high-precision detection of book spines is a critical requirement. However, the varying tilt angles and diverse aspect ratios of books on library shelves often reduce the effectiveness of conventional object detection algorithms. To address these challenges, this study proposes an enhanced oriented R-CNN algorithm for book spine detection. First, we replace the standard 3 × 3 convolutions in ResNet50’s residual blocks with deformable convolutions to enhance the network’s capacity for modeling the geometric deformations of book spines. Additionally, the PAFPN (Path Aggregation Feature Pyramid Network) was integrated into the neck structure to enhance multi-scale feature fusion. To further optimize the anchor box design, we introduce an adaptive initial cluster center selection method for K-median clustering. This allows for a more accurate computation of anchor box aspect ratios that are better aligned with the book spine dataset, enhancing the model’s training performance. We conducted comparison experiments between the proposed model and other state-of-the-art models on the book spine dataset, and the results demonstrate that the proposed approach reaches an mAP of 90.22%, which outperforms the baseline algorithm by 4.47 percentage points. Our method significantly improves detection accuracy, making it highly effective for identifying book spines in real-world library environments.

## 1. Introduction

As modern library collections continue to expand, the workload associated with manual on-shelf inventory management has increased substantially. Advances in computer vision technology, coupled with the decreasing cost of compact cameras, have prompted researchers to develop vision-based systems for the automatic detection and identification of on-shelf books. Leveraging computer vision for book localization and identification allows libraries to implement intelligent inventory management without incurring excessive costs, significantly enhancing operational efficiency and offering promising applications for the future. Since only the spines of books are visible to both the human eye and cameras in typical shelf images [1], the accurate detection of book spines is essential for effective book recognition. Book spine detection is a crucial task with significant practical applications in various fields, particularly in library systems, digital archiving, and inventory management. In modern libraries, the automation of book spine detection can greatly enhance the efficiency of book shelving, retrieval, and inventory tracking, reducing reliance on manual labor and minimizing errors. For digital archiving, detecting and recognizing book spines is an essential step in creating high-quality digital catalogs that enable users to search and access books remotely. Furthermore, in inventory management systems, accurate book spine detection ensures the timely updating of stock information, supports automated audits, and facilitates seamless integration with other library management technologies [2].

Traditional book spine detection typically relies on a combination of feature extraction and classification. Researchers, both domestically and internationally, have employed line detection algorithms, such as the Hough transform [3,4,5,6], to extract straight lines along the edges of book spines. Alternatively, some methods use Maximally Stable Extremal Regions (MSERs) for feature extraction to identify text regions on spines, enabling spine localization [7]. However, these conventional approaches rely heavily on handcrafted features, which are often limited in scope. Additionally, they depend on empirically set thresholds, resulting in low accuracy and poor generalizability across different scenarios.

Recently, deep learning has rapidly advanced, which revolutionized object detection, with Convolutional Neural Networks (CNNs) demonstrating superior efficiency and performance compared to traditional approaches [8,9,10,11,12,13,14]. Object detection detectors are broadly divided into two main types: one-stage and two-stage methods. One-stage detectors, such as the YOLO series, directly predict both the categories and bounding box coordinates of the objects in an image using a single convolutional network. These methods significantly improve detection speed, making them ideal for real-time applications [15,16,17]. On the other hand, two-stage methods, such as Faster R-CNN, preliminary identify the potential Region of Interest (RoI) through preset anchor boxes in the Region Proposal Network (RPN). Afterwards, these RoIs are classified, and their bounding boxes are refined through the detection head, making two-stage detectors better suited for tasks requiring high precision [18,19].

Library inventory management prioritizes accuracy over real-time detection speed. To address the challenge of detecting tilted book spines on shelves, this study explores an oriented object detection approach specifically designed for book spines. Detecting tilted spines presents several challenges, including the following:

- Tilted placement and overlapping bounding boxes: The varying angles of book spines, caused by differences in placement and camera orientation, complicate detection. When densely arranged tilted spines are detected using horizontal bounding boxes, each box may contain information from adjacent spines. This results in high Intersection over Union (IoU) values between neighboring bounding boxes, leading to incorrect filtering during Non-Maximum Suppression (NMS) [20].
- Varying aspect ratios: Spines exhibit different aspect ratios due to variations in height and thickness, with some slim spines having very high aspect ratios. Accurate angle prediction becomes more critical for detecting such targets [21].
- Geometric deformation: Book spines may appear distorted in captured images due to the angle of the camera. Detecting spines under these conditions requires robust feature extraction to handle irregular shapes effectively.

To address the challenges in book spine object detection, it is essential to enhance the model’s ability to locate tilted or deformed book spines through more robust feature extraction and feature fusion mechanisms. In this study, we propose an improved oriented R-CNN model to enhance detection accuracy for on-shelf books in library environments. Our network adopts a two-stage oriented object detection framework, leveraging deformable convolutions for feature extraction and incorporating a secondary feature fusion mechanism to improve model precision.

We applied data augmentation to the book spine dataset [22] and used it for both the objective and subjective performance evaluation of the model. Additionally, we performed qualitative visual evaluations using the images from the on-shelf-books-recognition dataset [23] to validate the robustness and generalizability of the proposed model in real library environments. The experimental results demonstrate that the proposed model can accurately detect on-shelf books in libraries and exhibits strong generalization capabilities. The key contributions of this study are as follows:

1. Deformable Convolutional Networks (DCNs) [24] are used to replace standard convolutions in the ResNet50 residual blocks, to handle geometric deformations and enhance the spine detection accuracy of the model.
2. The Path Aggregation Network (PANet) [25] is integrated into the neck structure to enhance feature fusion, strengthening the feature layer’s ability to localize book spines effectively.
3. An optimized method for selecting the initial cluster centers in the K-median algorithm is developed. This improved K-median algorithm is applied to the book spine dataset to adaptively calculate the anchor box aspect ratios, improving the model’s convergence speed and training performance.

The remainder of this paper is structured as follows: Section 2 provides a literature review on book spine object detection. Section 3 presents the architecture of the book spine detection detector along with the improvements made to the model. Section 4 details the experimental evaluation metrics, along with ablation studies and comparisons with other models. Section 5 summarizes the key findings of the paper and outlines potential directions for future research.

## 2. Related Work

In recent years, extensive research has focused on book spine detection for library on-shelf inventory management. Some studies have aimed at enhancing the performance of classical horizontal detectors, while others have explored various techniques to improve the accuracy of detecting tilted spines. This section provides a review and analysis of recent advancements in this field.

### 2.1. Horizontal Object Detection Methods

Book spine detection is a specialized object detection problem. Wang et al. [26] designed a book label detection network based on YOLOv3-Tiny, which leverages multi-scale output and skip connections to enhance the detection of small targets. However, this approach does not tackle the detection of elongated book spine targets. Xie et al. [27] developed a book information extraction algorithm using YOLOv5 combined with Optical Character Recognition (OCR). Their approach also employs a statistical pre-pruning strategy to improve YOLOv5’s detection speed. However, this method may not provide optimal detection accuracy for tilted books. Zhou et al. [23] utilized the Mask R-CNN network to extract spine masks from bookshelf images and applied affine transformations to obtain rectangular spine regions. Recognizing the limitations of Mask R-CNN’s reliance on horizontal bounding boxes, Wang et al. [28] addressed the challenge of detecting tilted spines by rotating input images at multiple angles before detection. However, this preprocessing step leads to a decrease in detection efficiency.

### 2.2. Oriented Object Detection Methods

Most existing object detection algorithms rely on horizontal bounding boxes, which are ill-suited for tilted and slim book spines. In contrast, oriented object detection networks predict angular information, enabling the more precise regression of rotated bounding boxes. Zeng et al. [20] introduced angular parameter regression into Mask R-CNN, enabling the detection of spines using oriented bounding boxes. While this method improves the accuracy of detecting densely tilted spines, it faces challenges with accuracy loss for upright spines due to the periodicity of the rotation angle.

These years, there have been various oriented detection models. In the area of one-stage oriented object detectors, Han et al. [29] introduced S2ANet, a lightweight structure that generates high-quality anchors and alignment features through alignment convolutions. Yang et al. [30] developed R3Det, which enhances detection by progressively refining predictions from coarse to fine granularity. Ultralytics extended the YOLOv8 OBB architecture to support bounding boxes with rotation angles, marking the first official YOLO series release with this capability, achieving an effective balance between speed and accuracy [31]. For two-stage oriented object detection, Jiang et al. [32] designed the Rotational Region Convolutional Neural Network (R2CNN), incorporating angle prediction into base Faster R-CNN and introducing a specialized NMS algorithm for rotated bounding boxes. Ma et al. [33] proposed the Rotated Region Proposal Network (RRPN), which uses rotated anchor boxes at multiple angles, but the dense anchor strategy results in high computational costs. Ding et al. [34] developed the RoI Transformer, which leverages fully connected layers to learn spatial transformations from horizontal to rotated bounding boxes, reducing redundant boxes but struggling with high aspect ratio targets. Xie et al. [35] developed the oriented R-CNN, which employs a midpoint offset representation for rotated candidate boxes. The oriented RPN using this representation can generate high-quality rotated boxes with lower computational costs.

While the above methods demonstrate promising performance, significant improvements are still needed for accurate book spine detection, particularly when dealing with geometric deformation and variations in the aspect ratio of book spines. This paper proposes a novel approach that integrates DCN and PANet to extract robust multi-scale features. Furthermore, clustering analysis is utilized to determine optimal aspect ratios for book spines, which can further enhance the model’s detection accuracy.

## 3. Methodology

**Figure 1:**
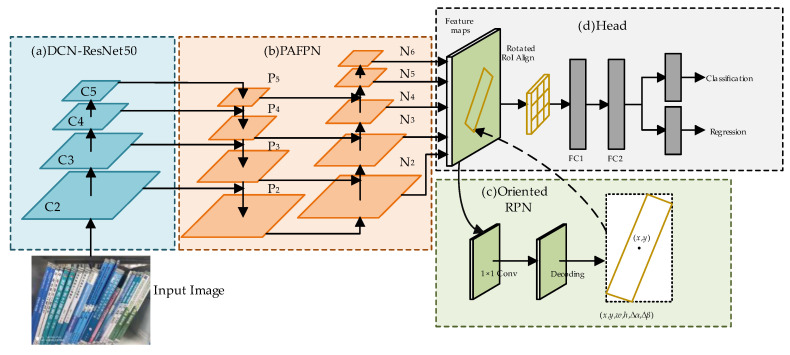
The architecture of the enhanced oriented R-CNN model.

To enhance the detection accuracy of book spines in bookshelf images, we enhance the basic oriented R-CNN network, and the overall structure of the optimized network is depicted in Figure 1. The network consists of the following key components: (a) backbone, (b) neck structure, (c) oriented RPN, and (d) detection head. The backbone processes input book images and uses ResNet50 with Deformable Convolution Networks (DCNs) for feature extraction, improving the ability to detect deformed or tilted spines. The neck structure integrates the Path Aggregation Feature Pyramid Network (PAFPN) to fuse multi-scale features, enhancing the model’s ability to localize objects at different scales. The oriented RPN generates rotated candidate boxes from horizontal anchors, accounting for the rotation angles. The rotated RoI alignment module then extracts feature vectors from these rotated boxes, ensuring that fixed-size features are obtained from the feature maps. Finally, the detection head takes the feature vectors and rotated candidate boxes as input and generates the model’s final predictions through fully connected layers.

### 3.1. Enhanced Backbone Network

**Figure 2:**
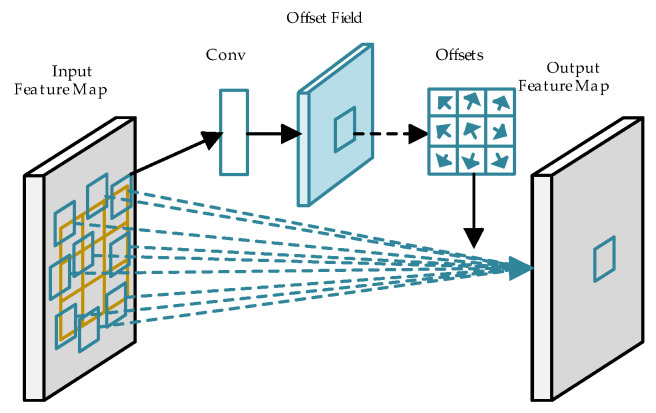
Schematic of deformable convolutional layer.

The book image is first processed through the backbone network for feature extraction. While ResNet50 typically employs standard convolution with fixed sampling locations, this approach is limited in the context of book spine detection. Due to the significant variation in the sizes and shapes of book spines, standard convolution is restricted by a fixed square receptive field and a rigid sampling pattern and lacks the capacity for long-range feature modeling, making it suboptimal for detecting elongated or tilted spines. To address these limitations, the backbone uses the DCN to improve feature extraction, allowing for better localization of book spines with varying shapes. Figure 2 illustrates the structure of a 3 × 3 DCN used within the backbone. The DCN introduces an offset learning mechanism that shifts the convolutional kernel’s sampling points on the input feature map. This offset allows the receptive field to move beyond its original square shape, dynamically aligning with the contours of the book spine. Even when the shape of a book spine is distorted due to variations in camera angle, the DCN’s adaptability ensures that the receptive field remains focused on the relevant spine regions. This flexibility enhances the network’s ability to accurately model and detect book spines under varying geometric conditions.

**Figure 3:**
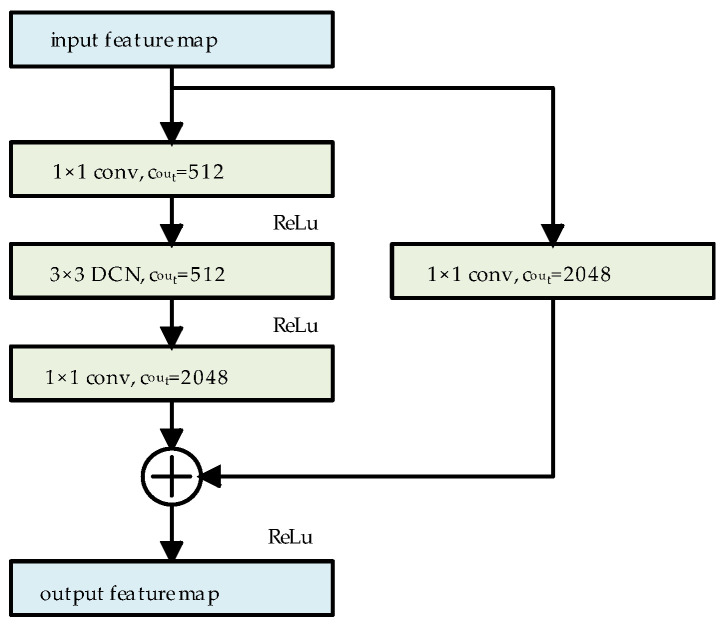
Schematic of deformable residual block structure.

We used the same architecture for the residual blocks as ResNet50. In the residual blocks of the last three layers of ResNet50, all 3 × 3 standard convolutional layers are replaced with 3 × 3 DCNs. Taking the last residual block as an example, the schematic structure of the residual block after this modification is presented in Figure 3. In this configuration, the DCN adaptively extracts features based on the shape of the target, while 1 × 1 convolution is employed to adjust the channel numbers in the feature map, where c_out_ denotes the output channel numbers of the convolution.

The DCN enhances flexibility by introducing offsets to each sampling point within the convolutional kernel, enabling random sampling near the current position rather than being confined to a fixed grid structure. This allows the network to better align with irregular target shapes. The mapping relationship between the output and input feature maps is shown in the following equation:

(1) $$Y(p_{0})=\sum_{p_{n}\in R}w(p_{n})\cdot X(p_{0}+p_{n}+\Delta p_{n}),$$

where *X* and *Y* represent the input and output feature maps, respectively, *w* represents the sampling weight of the DCN, *p*_0_ is the position of the feature point of *Y*, the set *R* defines the relative position of all the sampling points of the DCN, with *p_n_* as an element of *R*, and Δ*p_n_* represents the offset of the DCN. Since the offset Δ*p_n_* is typically a non-integer, the value of *X* at (*p*_0_ + *p_n_* + Δ*p_n_*) must be obtained through bilinear interpolation, as expressed in Equation (2).

(2) $$X(p)=\sum_{q}G(p,q)\cdot X(q),$$

where *p* = (*p*_0_ + *p_n_* + Δ*p_n_*) represents the position after applying the offset, *q* enumerates the four neighboring integer coordinate positions surrounding *p*, and *G* denotes the kernel function of bilinear interpolation.

### 3.2. Feature Fusion Module

**Figure 4:**
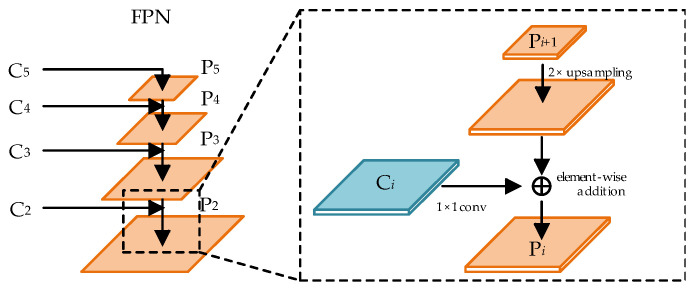
Schematic of FPN structure.

Let the set of feature maps output by the backbone network be {C_2_, C_3_, C_4_, C_5_}. As the CNN layers deepen, the receptive field of the feature maps increases, but their size and resolution decrease. As a result, high-level feature maps are better for detecting large objects, while low-level feature maps are more effective for detecting smaller ones. The traditional Feature Pyramid Network (FPN) follows a top-down feature fusion path, where the feature map C_5_ is first copied to generate P_5_. Subsequent feature fusion proceeds sequentially, as shown in Figure 4. Specifically, the high-level feature map P*_i_*_+1_ (from the previous stage) is progressively upsampled and added to the corresponding feature map C*_i_*, where *i* ∈ [2, 4]. In the proposed method, bilinear interpolation is used for the upsampling operation, providing more accurate sampling results than traditional nearest-neighbor interpolation.

**Figure 5:**
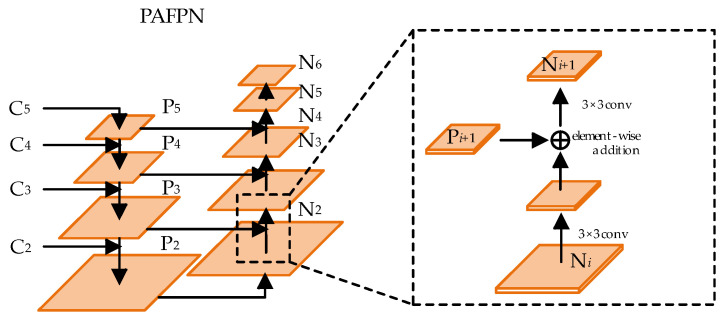
Schematic of PAFPN structure.

In book spine detection, the accurate localization of slim book spines is crucial. However, the feature maps P_4_ and P_5_ from the traditional FPN are limited by resolution, leading to a loss of detailed information for spine localization. To address this, we introduce a secondary feature fusion structure from PANet into the traditional FPN, resulting in the development of the Path Aggregation Feature Pyramid Network (PAFPN). The overall framework of PAFPN is depicted in Figure 5. The feature layers output by the FPN are denoted as {P_2_, P_3_, P_4_, P_5_}. A bottom-up path structure is incorporated into the FPN, wherein the feature maps from P_2_ to P_5_ are downsampled by a factor of 2 and then combined with the feature maps from the upper layers. This process generates a new set of feature map combinations denoted as {N_2_, N_3_, N_4_, N_5_, N_6_}, where N_2_ is derived from the replication of P_2_, and N_6_ denotes the result of applying max pooling to reduce the size of N_5_ by half.

The specific process of the bottom-up path is illustrated in the dashed box in Figure 5. This process involves laterally connecting the higher-resolution feature map N*_i_* to the lower-resolution feature map P*_i_*_+1_ to produce a new feature map N*_i_*_+1_. Initially, N*_i_* is passed through a 3 × 3 convolutional layer with a stride of 2 to reduce its dimensions by half. Subsequently, feature fusion is performed through element-wise addition with the feature map P*_i_*_+1_. Finally, another 3 × 3 convolutional layer is applied to produce N*_i_*_+1_ for further fusion processing. This bottom-up secondary feature fusion path facilitates the transfer of precise localization information from the lower layers to the higher layers, addressing the information loss in the higher-level feature maps. Consequently, this approach enhances the network’s localization performance for detecting book spine targets.

### 3.3. Oriented RPN

In the oriented RPN, feature maps from the PAFPN, denoted as {N_2_, N_3_, N_4_, N_5_, N_6_}, are associated with a predefined set of anchor box areas: {32^2^, 64^2^, 128^2^, 256^2^, 512^2^}. For each anchor point, three aspect ratios (defaulting to {0.5, 1.0, 2.0}) are used to generate horizontal rectangular anchor boxes. These boxes are then processed through a 3 × 3 convolutional layer followed by two 1 × 1 convolutional layers for classification and regression, generating the initial rotated candidate boxes.

**Figure 6:**
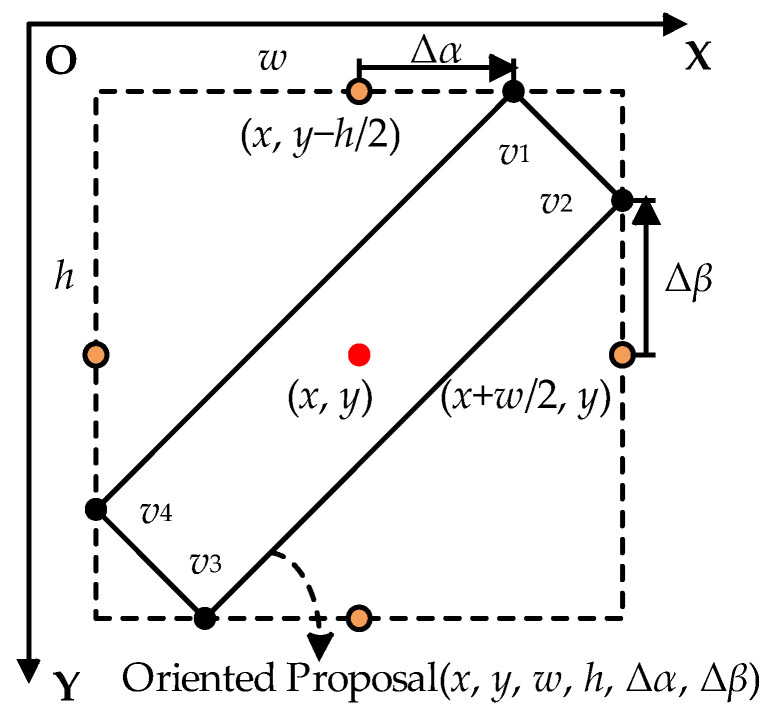
Diagram of midpoint offset representation.

During the computation of the rotated candidate boxes, the midpoint offset method proposed by the oriented R-CNN is employed. This method is recognized for its efficiency and accuracy compared to the box generation methods used in other oriented object detection networks. Each anchor box is represented by (*x_a_*, *y_a_*, *w_a_*, *h_a_*), where *x_a_* and *y_a_* represent the coordinates of the anchor box’s center, while *w_a_* and *h_a_* denote its width and height, respectively. The midpoint offset representation of the rotated candidate box is shown in Figure 6, where the rotated box is expressed as (*x*, *y*, *w*, *h*, Δ*α*, Δ*β*). Here, (*x*, *y*, *w*, *h*) denotes the horizontal external rectangle of the rotated box, and the coordinates of the four vertexes of the rotated box are given as {*v_1_*, *v_2_*, *v_3_*, *v_4_*}, Δα denotes the offset of *v_1_* relative to the midpoint of the top edge of the horizontal box, while Δ*β* indicates the offset of *v_2_* relative to the midpoint of the right side of the horizontal box.

In this approach, the oriented RPN regresses the offset *δ* = (*δ_x_*, *δ_y_*, *δ_w_*, *δ_h_*, *δ_α_*, *δ_β_*) of each predefined horizontal anchor box through a dedicated convolutional layer. This offset describes the adjustments needed in the position, size, and rotation of the rotated candidate box. The rotated boxes are generated by decoding the regressed offset using Equation (3).

(3) $$\left\{\begin{array}{ll} x=\delta_{x}\cdot w_{a}+x_{a},\; & y=\delta_{y}\cdot h_{a}+y_{a}\\ w=w_{a}\cdot e^{\delta_{w}},\; & h=h_{a}\cdot e^{\delta_{h}}\\ \Delta \alpha =\delta_{\alpha }\cdot w,\; & \Delta \beta =\delta_{\beta }\cdot h \end{array}\right.,$$

where (*x_a_*, *y_a_*, *w_a_*, *h_a_*) represents the horizontal anchor box and (*x*, *y*, *w*, *h*, Δ*α*, Δ*β*) represents the calculation results of the rotated candidate box.

In the detection head, the rotated candidate boxes generated by the oriented RPN are further fine-tuned through fully connected layers. The final rotated bounding boxes then ensure precise alignment with the tilted book boundaries.

The default set of anchor box aspect ratios in the oriented RPN is {0.5, 1.0, 2.0}. However, these ratios significantly differ from the aspect ratios of elongated book spines, resulting in low Intersection over Union (IoU) values between the anchor boxes and the training set samples. This misalignment makes it challenging to accurately match positive samples during model training, thereby slowing convergence and reducing training efficiency. Since the oriented RPN matches anchor boxes to the horizontal external rectangles of ground truth bounding boxes, it is essential to perform a clustering analysis on the horizontal bounding boxes of all samples in the training set to optimize the anchor box design.

### 3.4. Anchor Boxes Optimization Algorithm

**Figure 7:**
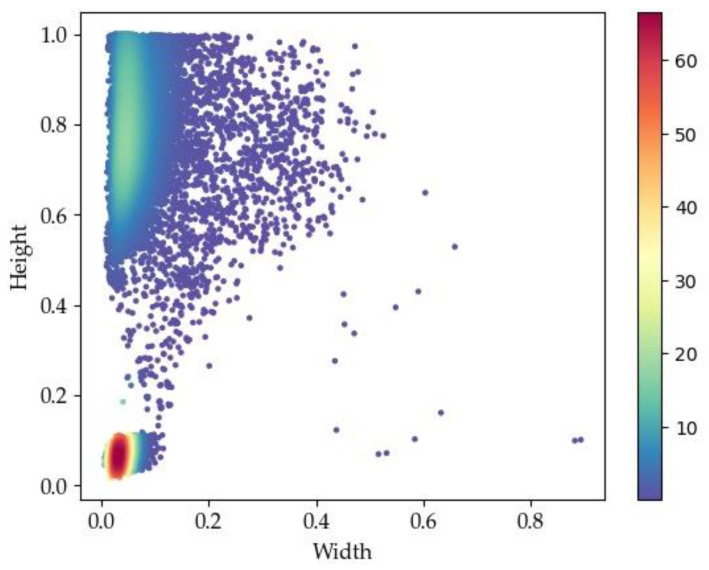
Scatter density plot of the book spine detection dataset.

To address the issue of poor alignment between anchor boxes and the dataset, an anchor box clustering algorithm is employed. Common algorithms for this purpose include K-means and K-median clustering. To select an appropriate clustering algorithm based on the dataset characteristics, we visualize the book spine detection dataset. The scatter density plot of the dataset is shown in Figure 7.

In this plot, the horizontal axis denotes the width and the vertical axis denotes the height. The color bar on the right indicates the density of the scatter points. The color transitions from cool tones (e.g., blue) to warm tones (e.g., red), representing a change in density from low to high. Specifically, warmer colors indicate areas with a higher point density, while cooler colors indicate sparser regions. The data points are primarily concentrated in the upper-left and lower-left regions, with some outliers distributed in the right half of the plot.

During the iteration of clustering algorithms, the cluster centers need to be continuously updated. K-means recalculates the center of each cluster by averaging the data points within the cluster. However, when the data points exhibit tight clusters with a few distinct outliers, using the mean to determine the cluster center can lead to instability. The center of clusters with outliers becomes easily influenced by the outliers, resulting in less accurate clustering. K-means relies on the mean distance to assign cluster centers, making it sensitive to outliers. In contrast, K-median uses the median distance, which is more robust against the influence of anomalous data points. Therefore, the K-median clustering is selected to cluster the bounding boxes of the dataset.

A known limitation of the K-median algorithm is that it randomly selects initial cluster centers and poor initial cluster centers can cause the algorithm to converge to a local optimum. To overcome this issue, a refined K-median algorithm is developed to adaptively refine the initialization of cluster centers, enhancing convergence and clustering performance. Algorithm 1 describes the overall process.
**Algorithm 1** Improved K-median**Input**: DATA S; clusters *K*.**Output**: C = {box_1_,box_2_,...,box*_k_*}.Randomly select the first candidate cluster center from S, number of current candidate clusters K* = 12.while K* < *K* do3.Calculate the distance *d*(*i*) between each sample *i* in S and its nearest cluster center4.The sample *j* corresponding to the maximum value of *d*(*i*) (*j* = argmax(*d*(*i*))) is selected as the next cluster center, then K* = K* + 15.Recalculate the distance from each sample *i* to the K* centers and assign it to the closest class6.Reset the cluster center according to the median distance of the samples within the class7.end while8.Execute the standard K-median algorithm with the candidate centers as the initial cluster centers to obtain the precise *K* cluster centers.

During the iterative process of initializing the cluster centers, the algorithm selects the sample with the greatest distance from the existing cluster centers as the next center. Subsequently, it recalculates each cluster center based on the median distance of the samples within the corresponding class. This strategy enhances the separation between clusters during initialization while minimizing the influence of outliers on the clustering process.

The distance *d* between samples is evaluated using the IoU metric, as defined in Equation (4).

(4) $$d{(\mathrm{box}}_{i}{,\mathrm{box}}_{j})={1-\mathrm{IoU}(\mathrm{box}}_{i}{,\mathrm{box}}_{j}),$$

where box*_i_* and box*_j_* represent different bounding box samples, and IoU(box*_i_*, box*_j_*) represents the Intersection of Union between these two samples. The calculation of IoU is shown in Equation (5).

(5) $${\mathrm{IoU}(\mathrm{box}}_{i}{,\mathrm{box}}_{j})=\frac{{\mathrm{box}}_{i}\cap {\mathrm{box}}_{j}}{{\mathrm{box}}_{i}\cup {\mathrm{box}}_{j}},$$

where “∩” denotes the area of overlap between the two samples and “∪” denotes the area of their union.

To optimize anchor box aspect ratios for book spine detection, we apply the proposed K-median algorithm to cluster the dataset and analyze the results. For the input bounding box, the algorithm generates *K* cluster centers, each represented as (*w*, *h*). These centers, with high IoU values relative to the dataset, are used to calculate aspect ratios, which then serve as anchor aspect ratio hyperparameters for the oriented RPN stage. Subsequent experiments will assess the impact of these cluster-derived anchor ratios on training performance and detection accuracy.

## 4. Experiments and Results

### 4.1. Dataset

**Figure 8:**
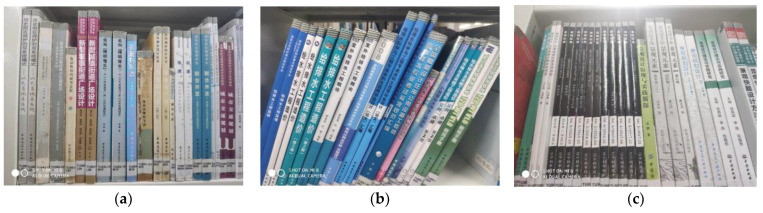
Samples of book spine dataset: (**a**) near-vertical image; (**b**) tilted books; (**c**) low-angle shot image.

The datasets used in this study are the book spine dataset [22] and on-shelf-books-recognition dataset [23]. The book spine dataset [22] was mainly used for the experiments. This dataset comprises 661 images of library shelves, captured using smartphones at Shenzhen University. The images include various shooting angles and a range of book tilt angles, with 283 featuring tilted books (mainly caused by camera angles and book placement) and 378 near-vertical images (tilt angle less than 5°). Sample images from the book spine dataset are shown in Figure 8.

The original dataset contains 15,454 manually labeled book spine instances, with an average of 23 spines per image. Since both the text on the spine and the call number on the book label are essential for book identification, this study considers the spine and the label as two categories of targets for detection. Polygonal annotations for book labels were added using the Labelme tool, and the annotations were converted to the DOTA dataset format to facilitate model training.

**Figure 9:**
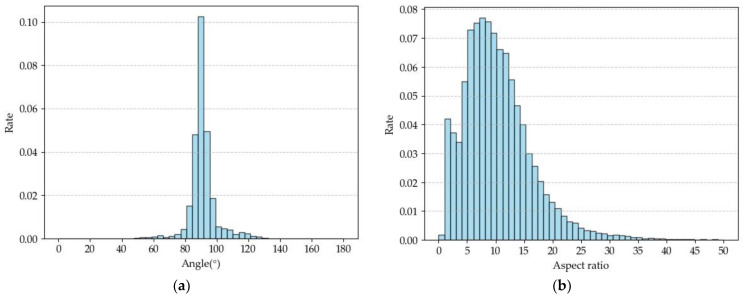
Dataset distribution: (**a**) angle distribution histogram; (**b**) aspect ratio distribution histogram.

Figure 9 shows the distribution of object rotation angles and aspect ratios in the dataset. The rotation angle, defined as the angle between the longer side of the bounding box and the positive x-axis, ranges from [0°, 180°). Most angles fall within 45° to 135°, with the highest concentration near 90°, indicating that books are predominantly placed vertically but exhibit varying tilt orientations. The aspect ratios span a broad range, reflecting significant variations among book spines. A clustering analysis will be conducted on these bounding boxes to derive aspect ratio hyperparameters for the oriented RPN, aiming to enhance the model’s training performance.

**Figure 10:**
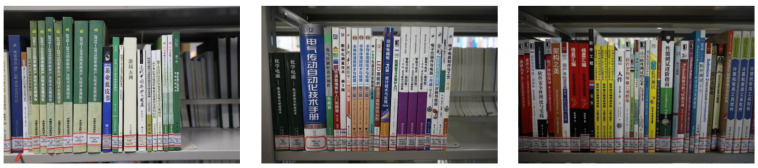
Samples of on-shelf-books-recognition dataset.

The on-shelf-books-recognition dataset [23], collected from the National Agricultural Library of CAAS, contains 425 bookshelf images with a resolution of 1920 × 1080. Figure 10 shows sample images from the dataset. As the images lack manual annotations, this dataset is used for the qualitative evaluation of the model’s generalization. The model is trained and evaluated on the book spine dataset [22], and its detection results are qualitatively assessed on the on-shelf-books-recognition dataset [23] to demonstrate its robustness and generalizability.

### 4.2. Book Spine Data Augmentation

**Figure 11:**
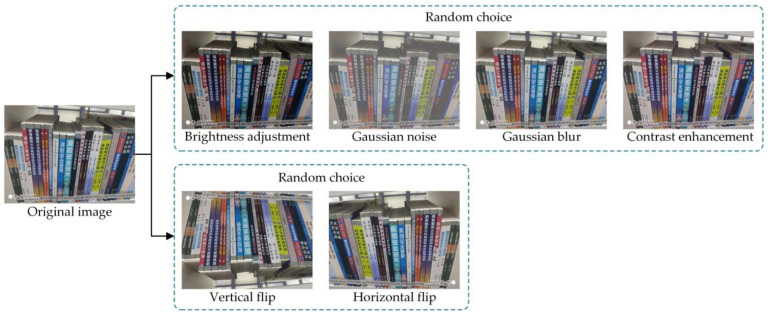
Illustration of data augmentation.

In addition, to expand the dataset and enhance its diversity, data augmentation techniques were applied. The data augmentation process, illustrated in Figure 11, involves the following steps:

1. Brightness Adjustment: Image brightness is randomly adjusted using a factor *γ*, uniformly sampled from [0.5, 1.5]. This simulates varying lighting conditions in libraries, improving detection robustness under diverse environments.
2. Adding Gaussian Noise: Gaussian noise is generated using the formula *n*∼*N*(*μ*, *σ*^2^), where the mean *μ* = 0 and variance *σ*^2^ = 0.05, and then added to the images. This step mimics noise caused by image sensors or environmental interference, enhancing the model’s robustness to noisy inputs.
3. Adding Gaussian Blur: The images are blurred using a Gaussian filter with a kernel size of 17 × 17 and a standard deviation of 0. This simulates blurring due to camera focus issues or motion, helping the model learn to detect book spines under blurred conditions.
4. Contrast Adjustment: Contrast is enhanced using histogram normalization. By calculating the maximum (*I*_max_) and minimum (*I*_min_) pixel intensity values of the input image, the intensity range is linearly mapped to [0, 255]. This adjustment improves the visual distinction between book spines and their backgrounds, simulating variations in contrast caused by different cameras or shooting environments.
5. Random Flipping: Images are randomly flipped horizontally or vertically. This increases the diversity of training samples and simulates unexpected viewpoint variations during image acquisition.

These transformations simulate environmental variations encountered in real-world library settings, enhancing the model’s generalization capability and robustness. To prevent data leakage between the training and test sets, the original 661 images were first randomly split into training and test sets at an 8:2 ratio. Data augmentation was applied separately to both subsets. After augmentation, the total number of images increased to 1983, with the training set containing 1587 images and the test set containing 396 images. These expanded datasets were used for subsequent experiments.

### 4.3. Experimental Evaluation Index

We employ average precision (AP) and mean average precision (mAP) as key metrics of the model’s detection accuracy, with their respective formulas provided in Equations (6)–(9). Additionally, model complexity is quantified by counting up the total number of parameters, measured in megabytes (MB).

(6) $$P=\frac{\mathrm{TP}}{\mathrm{TP}+\mathrm{FP}},$$

(7) $$R=\frac{\mathrm{TP}}{\mathrm{TP}+\mathrm{FN}},$$

(8) $$\mathrm{AP}=\int_{0}^{1}P(R)\mathrm{dR},$$

(9) $$\mathrm{mAP}=\frac{1}{n}\sum_{i=1}^{n}{\mathrm{AP}}_{i},$$

where, in Equation (6), precision (P) represents the ratio of rightly identified positive samples to the total number of predicted positive samples. In Equation (7), recall (R) is defined as the ratio of rightly identified positive samples to the total number of ground truth samples; true positives (TPs) refer to ground truth samples rightly identified as positive, false positives (FPs) refer to samples wrongly predicted as positive, and false negatives (FNs) refer to ground truth samples incorrectly predicted as negative. In Equation (8), average precision (AP) is the integral of the precision–recall curve over the interval [0,1] for a given IoU threshold. In Equation (9), mean average precision (mAP) is the average AP of all target categories. Let *n* denote the total number of classes. For the book spine detection dataset, *n* = 2, corresponding to the book spine and book label classes. In subsequent experiments, the IoU threshold for calculating AP and mAP is set to 0.75 to ensure a rigorous evaluation of detection accuracy.

### 4.4. Experimental Setup and Parameter Configuration

The experiments were performed using the PyTorch 1.13.0 and Python 3.9, with model training performed on a server equipped with an NVIDIA A800 GPU. The ResNet50 model, pre-trained on the ImageNet dataset, provided the initial weights for the training process. All input images were uniformly resized to 1024 × 1024 pixels before being fed into the network. To evaluate the effects of batch size, learning rate, and training epochs on the model’s performance, we designed hyperparameter experiments on the book spine dataset. The evaluation metric is mAP, with GPU memory usage recorded for each configuration. Each variant uses the Stochastic Gradient Descent (SGD) optimizer. We tested batch sizes of 2, 8, and 16. For a batch size of 2, the learning rate was set to 0.005. As the batch size increased, the learning rate was adjusted according to the square root scaling rule, ensuring proportional scaling to maintain training stability and performance. As shown in Table 1, with the same number of training epochs, a batch size of 2 and a learning rate of 0.005 achieved the highest mAP while using the least GPU memory. For a batch size of 16, increasing the training epochs from 48 (Variant 3) to 96 (Variant 4) improved the mAP from 79.86% to 80.20%, but the performance remained below that of Variant 1.

Based on the above experiments and analysis, the hyperparameter settings are as follows: an initial learning rate of 0.005, momentum of 0.9, weight decay of 0.0001, and a batch size of 2. The training lasted for 48 epochs, with the learning rate reduced to 10% of its previous value at epochs 32 and 44. This configuration was used to ensure stable convergence and optimal model performance.

### 4.5. Result and Discussion

#### 4.5.1. Bounding Boxes Analysis

The effectiveness of the proposed enhanced K-median clustering algorithm is evaluated by calculating the Mean Intersection over Union (mIoU) between the cluster centers and all sample instances. The mIoU is a metric for evaluating the quality of the cluster centers, as defined in Equation (6). A higher mIoU indicates a better alignment between the cluster centers and the underlying distribution of the data, thereby reflecting more accurate anchor box configuration for model training.

(10) $$\mathrm{mIoU}=\underset{i\in n,j\in K}{\mathrm{mean}}{\mathrm{IoU}(\mathrm{box}}_{i}{,\mathrm{centroid}}_{j})$$

**Figure 12:**
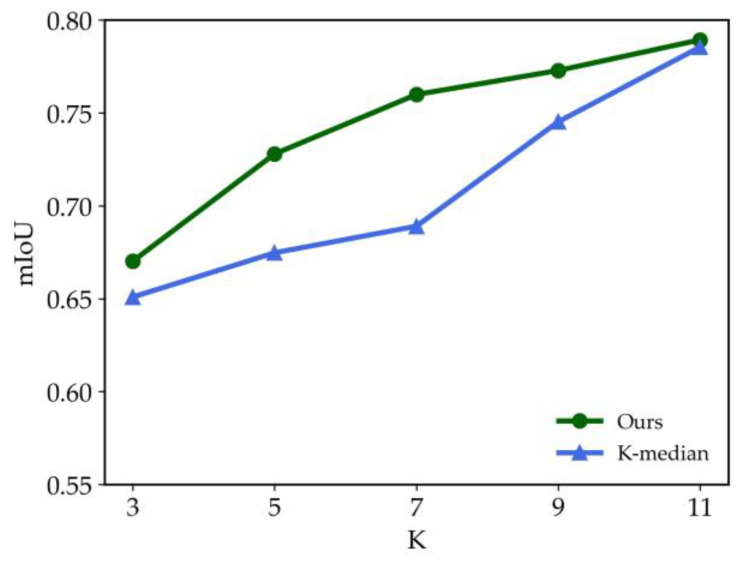
Comparison of different clustering methods.

To standardize the evaluation process, the width and height parameters (*w*, *h*) of all horizontal bounding boxes are normalized by dividing them by the image’s width and height. This ensures that both the sample widths and heights are unitless and scaled between 0 and 1. The book spine detection dataset is then subjected to clustering analysis using two different algorithms. The clustering performance is assessed using the mIoU metric, which measures the alignment between the cluster centers and the data distribution. Figure 12 presents the mIoU values achieved by the original algorithm and the proposed improved K-median algorithm across different numbers of cluster centers *K* (with *K* = 3, 5, 7, 9, 11). As shown, the improved K-median algorithm consistently yields higher mIoU scores, demonstrating its superior ability to align with the dataset’s characteristics and confirming the effectiveness of the proposed clustering approach.

**Figure 13:**
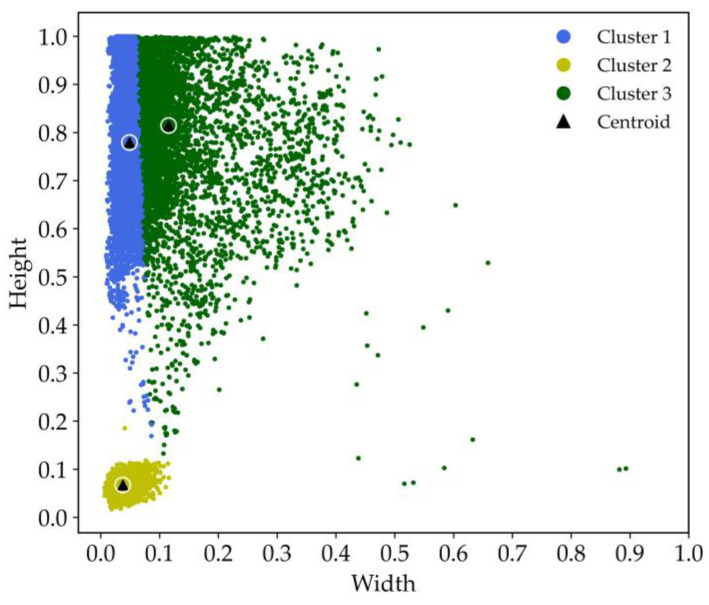
K-median clustering result of the book spine dataset.

The scatter plot generated by the improved K-median algorithm for *K* = 3 is presented in Figure 13. The cluster centers, selected by the improved K-median algorithm, are strategically located in areas with a high sample density, indicating strong cohesion within each cluster. This distribution demonstrates the reliability of the clustering results and highlights the algorithm’s ability to identify meaningful groupings within the dataset.

The clustering results are used to determine suitable anchor box aspect ratios for the book spine detection task. Since the oriented R-CNN framework uses three anchor box aspect ratios by default, *K* is set to 3 during the clustering process. The improved K-median algorithm identifies three cluster centers with the following width and height parameters: (0.037, 0.067), (0.116, 0.814), and (0.048, 0.779). From these, the aspect ratios (*h*/*w*) are calculated as follows: {1.79, 7.05, 15.94}. These calculated aspect ratios are then used as the anchor box aspect ratios for the oriented R-CNN network, ensuring that the anchor boxes closely match the shapes of book spines in the dataset. This customization improves the network’s detection accuracy by better aligning the anchor boxes with the objects of interest.

#### 4.5.2. Comparison of Anchor Boxes Aspect Ratios

The selection of the number of anchor box aspect ratios, denoted by *K*, is first explored in this experiment. Using the improved K-median algorithm, three sets of anchor box parameters were generated for the training set with *K* = 3, 5, and 7, respectively. Each set of aspect ratios was used as hyperparameters for training and testing the base oriented R-CNN model, with their performance compared against the baseline model trained using the default aspect ratios of {0.50, 1.00, 2.00} (*K* = 3, without clustering). The AP and mAP results from these experiments are given in Table 2.

Comparing rows 1 and 2 of Table 2, the performance of the model using the clustering algorithm for *K* = 3 yields no significant change in the AP for book labels compared to the baseline model with default aspect ratios. However, the AP for book spines improves by 10.16 percentage points, which increases the overall mAP by 5.07 percentage points. The default anchor box aspect ratios do not align well with the dimensions of book spines, while the ratios obtained through clustering significantly improve fitness between the anchor boxes and the book spines, leading to more effective detection. Increasing the *K* value beyond 3 (e.g., *K* = 5, 7) does not yield further performance improvements, indicating that the anchor box aspect ratios obtained with *K* = 3 already adequately represent the data distribution. This demonstrates that *K* = 3 is an optimal choice, as it captures the variability in book spine dimensions while avoiding unnecessary complexity. Thus, the aspect ratios {1.79, 7.05, 15.94}, derived from *K* = 3, are selected as the default ratios for subsequent experiments.

**Figure 14:**
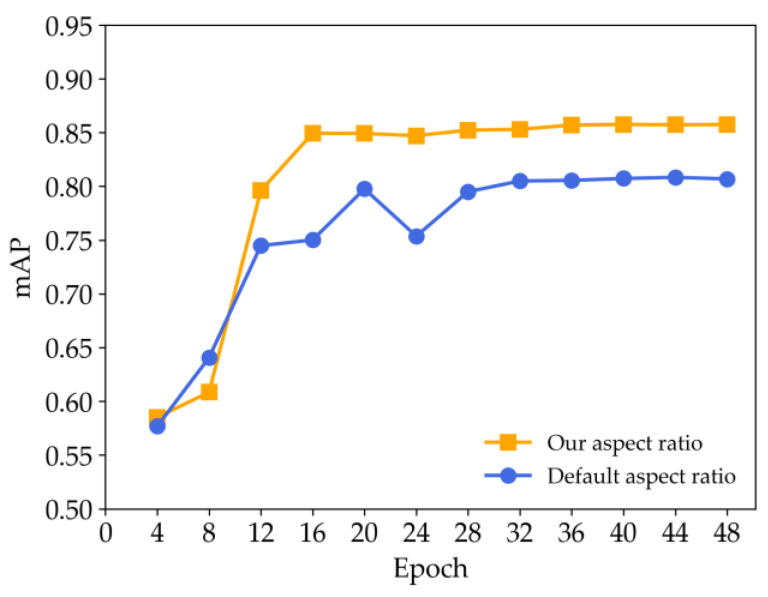
The mAP curves of model training under different aspect ratios.

Figure 14 presents the mAP curves for two models: one trained with the default aspect ratios {0.50, 1.00, 2.00} and the other with the aspect ratios derived from clustering {1.79, 7.05, 15.94}. Both models are trained for 48 epochs, with evaluations conducted every 4 epochs. The model with default aspect ratios converges around epoch 32, whereas the model trained with the clustered aspect ratios converges earlier, at around epoch 24. The clustered aspect ratio model not only converges faster but also consistently achieves higher accuracy throughout training, demonstrating better generalization and fitting to the book spine detection task. These results confirm the effectiveness of the clustering-based selection of anchor box aspect ratios, which improves the accuracy and training efficiency of the model for book spine detection.

#### 4.5.3. Ablation Studies

To assess the contribution of individual components in the proposed algorithm to the overall model performance, ablation experiments were conducted. These experiments progressively incorporate the clustering-based anchor box aspect ratios, DCN, and PAFPN structure into the oriented R-CNN model, evaluating their combined and individual effects on detection performance. The results are summarized in Table 3, where a “√” denotes that the corresponding module is included, and a “×” denotes that it is not.

A comparison between method 1 and method 2 reveals that the anchor box aspect ratios derived from clustering align more effectively with book spine and label targets than the default parameters. This adjustment significantly enhances the model’s training performance, resulting in a 5.07 percentage-point improvement in mAP. Building on the clustered anchor ratios used in method 2 (the baseline), method 3 replaces standard convolutions in the ResNet50 residual blocks with the DCN. This substitution increases the model’s feature extraction flexibility, yielding an additional 4.31 percentage-point gain in mAP. In method 4, the PAFPN structure is introduced to replace the traditional FPN. This modification improves the network’s ability to represent multi-scale features, which leads to a 4.40 percentage-point improvement in mAP, as it better captures both fine details and larger-scale contexts. Finally, method 5, representing the proposed algorithm, integrates both the DCN and PAFPN with the clustered anchor ratios. This combination delivers further performance gains, surpassing both method 3 and method 4 individually. The combination of the DCN and PAFPN increases the complexity of model training. Therefore, under the same hyperparameter settings, the combined improvement is minimal compared to using each module separately. Overall, the proposed algorithm improves the mAP by 4.47 percentage points compared to the baseline model, though it introduces a 10.02% increase in model parameters.

**Figure 15:**
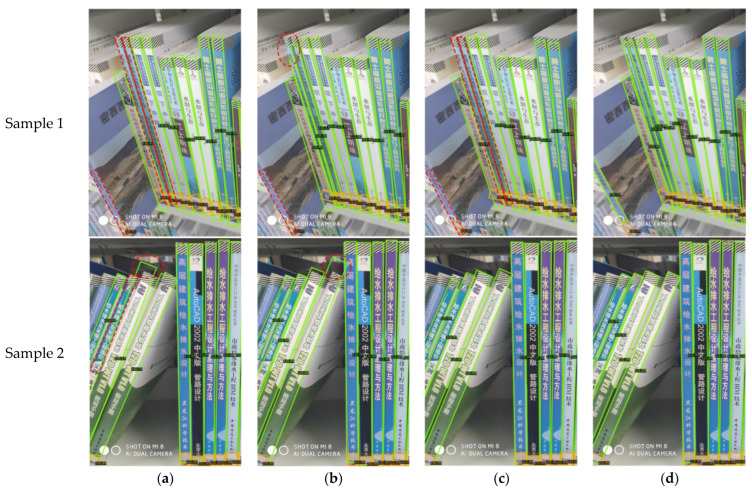
Comparison of detection results on book spine dataset between (**a**) method 2 (baseline), (**b**) method 3 (baseline + DCN), (**c**) method 4 (baseline + PAFPN), and (**d**) method 5 (baseline + DCN + PAFPN). In each detection result, the green boxes represent the book spine, the orange boxes denote the book label, and the red dashed ovals and boxes highlight the detection issues.

To visually illustrate the effectiveness of the DCN and PAFPN, methods 2–5 from Table 3 were applied to two test images for comparison. As shown in Figure 15, the left-side books of sample 1 were deformed due to the camera angle. Using standard convolutional layers, the baseline and method 4 (baseline + PAFPN) miss detections, while method 3 (baseline + DCN) improves detection. Method 5 (baseline + DCN + PAFPN) achieves the best results, demonstrating that the DCN enhances the detection of deformed objects; for sample 2, the baseline and method 3 (baseline + DCN) show inaccurate localization for tilted spines, whereas method 4 (baseline + PAFPN) and method 5 (baseline + DCN + PAFPN) provide more precise localization, highlighting the PAFPN’s contribution to improving localization accuracy.

In summary, our model effectively enhances the accuracy of book spine detection by combining multiple architectural improvements. Each module contributes independently to the overall performance, and their integration maximizes the model’s detection capabilities. The results demonstrate that our approach offers a balanced trade-off between accuracy and model complexity, providing superior detection performance for book spines and labels.

#### 4.5.4. Comparison with Previous Work

To assess the detection performance of the proposed algorithm, experiments were carried out with existing oriented object detection methods. The selected algorithms include one-stage detectors: R3Det [30], S2Anet [29], and YOLOv8n-OBB [36], as well as two-stage detectors: Rotated Faster R-CNN [37], RoI Transformer [34], oriented R-CNN [35], and STD-O [38]. To ensure a fair comparison, the anchor box aspect ratios of all anchor-based algorithms were set according to the results obtained in Section 4.5.2. The results are shown in Table 4.

Table 4 indicates that in comparison to the one-stage algorithms, the mAP of the proposed algorithm is 27.05 percentage points higher than R3Det, 16.17 percentage points higher than S2Anet, and 8.90 percentage points higher than YOLOv8n-OBB. And compared to the two-stage algorithms, its mAP is 23.80 and 22.45 percentage points higher than that of the Rotated Faster R-CNN and RoI Transformer, respectively.

The proposed algorithm and the STD-O algorithm both adopt the midpoint offset representation of the rotated bounding box introduced by the oriented R-CNN, which avoids the complex computation of the rotation angle and reduces the error caused by angle prediction, resulting in significantly higher mAP values for these three methods compared to others. Among them, STD-O uses a Vision Transformer (ViT) backbone instead of ResNet50 and performs decoupled parameter prediction at the detection head, achieving a higher accuracy than the oriented R-CNN. The proposed algorithm further improves the quality of feature extraction and feature fusion by improving both the backbone and neck networks, yielding higher mAP values than STD-O. Compared to the baseline model, the proposed algorithm improves the mAP by 4.47 percentage points, with the AP of book spine targets improving by 8.79 percentage points, achieving the best accuracy on the book spine detection dataset.

**Figure 16:**
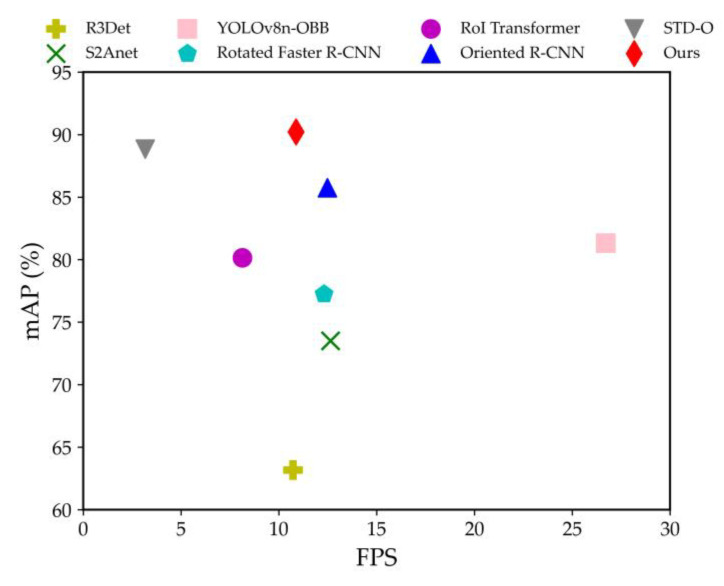
Comparison of model inference performance.

In terms of model complexity, YOLOv8n-OBB benefits from a lightweight network design, resulting in a minimal model size and computational load, achieving the fastest inference speed. Figure 16 visually compares the inference performance of the proposed algorithm with that of other methods. Although the inference speed of our method is slower than YOLOv8n-OBB, it is comparable to the speeds of the two single-stage detectors, R3Det and S2Anet. The proposed algorithm achieves the highest accuracy at the cost of additional model parameters. Therefore, to facilitate deployment on embedded and mobile devices, future work will focus on optimizing the model through pruning, distillation, and quantization techniques.

**Figure 17:**
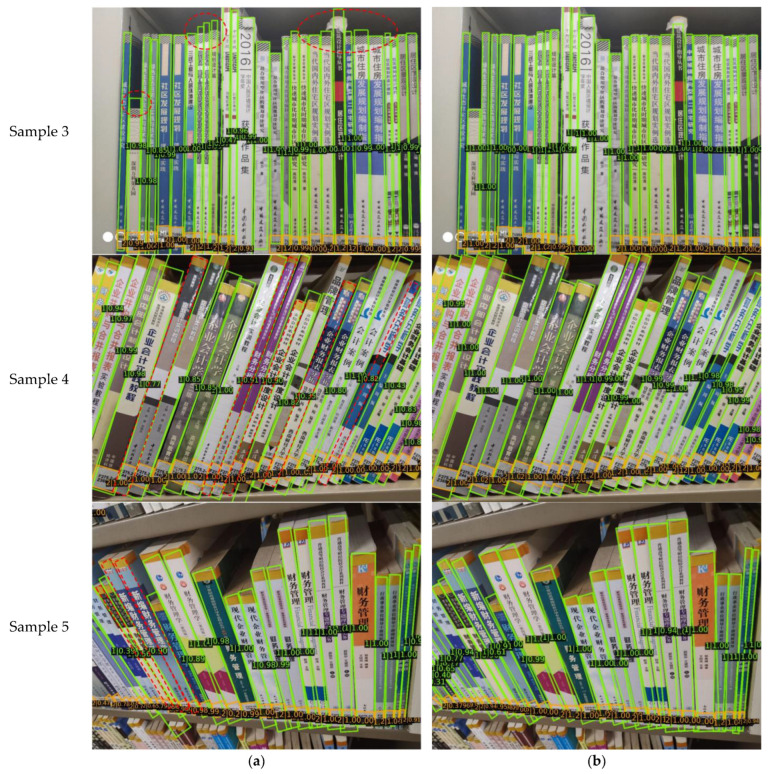
Comparison of detection results on book spine dataset between (**a**) oriented R-CNN and (**b**) improved oriented R-CNN. In each detection result, the green boxes represent the book spine, the orange boxes denote the book label, and the red dashed ovals and boxes highlight the detection issues.

Figure 17 illustrates the detection results of both the proposed algorithm and the baseline oriented R-CNN in three representative scenarios: vertically placed book spines, tilted book spines, and book spines captured from a top-down angle, highlighting the superiority of the proposed method. In Figure 17a, the detection results of the baseline are presented, while Figure 17b shows the results of the proposed algorithm. Each detection box is labeled with its predicted class (1 for book spine and 2 for book label) and confidence score, with red dashes marking key detection issues of the baseline algorithm. For vertical spines, the oriented R-CNN algorithm shows a deviation in localizing the upper boundary, whereas the proposed algorithm accurately aligns with the true boundary. For tilted spines, due to the high similarity among identical copies of the same book, the oriented R-CNN fails to detect five book spines. In contrast, the proposed algorithm, with enhanced feature extraction, avoids these missed detections and achieves higher confidence scores for detected spines. For book spines deformed by shooting angles, particularly those along the left edge of the image, the oriented R-CNN struggles to detect targets due to the angle-induced distortion. However, the proposed algorithm, leveraging the DCN’s robust feature extraction, successfully detects these spines.

**Figure 18:**
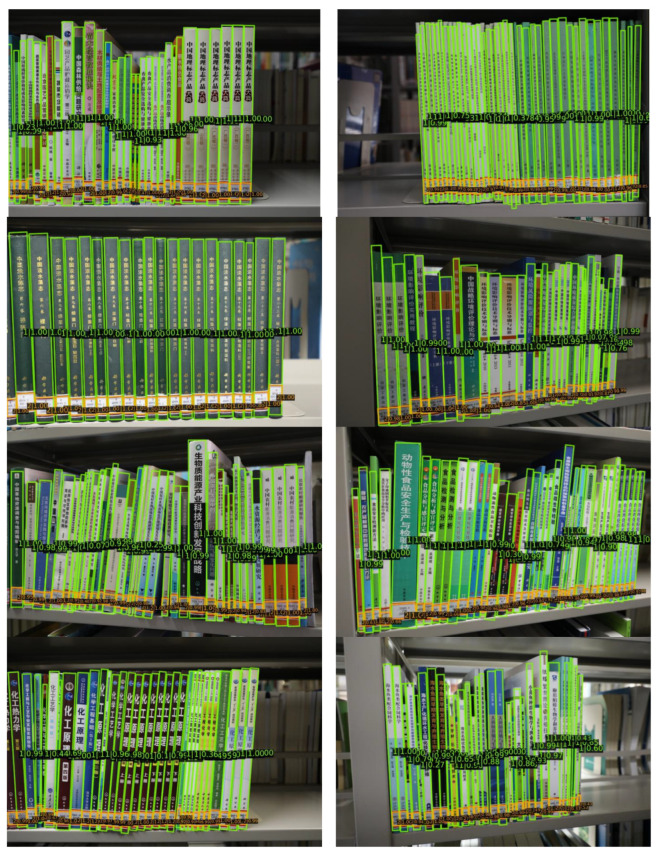
Visualization results on on-shelf-books-recognition dataset using improved oriented R-CNN.

To further assess the model’s generalization ability, we conducted a subjective evaluation on the on-shelf-books-recognition dataset [23], with the visualization of the detection results shown in Figure 18. Compared to the book spine dataset [22], these images have darker lighting and more complex backgrounds. Although the model was not trained on this dataset, its robust generalization is supported by data augmentation and effective feature extraction and fusion mechanisms. As a result, the model reliably detects book spines in these challenging scenarios, demonstrating its effectiveness for real-world library environments and confirming its practical applicability.

In summary, the proposed algorithm offers more accurate predictions of book spine boundaries in comparison to the baseline model, achieves higher recall for tilted and densely packed books, and reduces missed detections. These improvements demonstrate the superiority of the proposed model in processing different detection scenarios and enhancing overall accuracy.

## 5. Conclusions

This study addresses the challenge of the diverse aspect ratios and tilt angles of book spine targets, which negatively affect the accuracy of existing object detection algorithms. To overcome this, we propose an enhanced oriented R-CNN model specifically designed for the detection of book spines and book labels. We use the DCN in the ResNet residual blocks to enhance feature extraction capabilities. Additionally, we optimize the feature fusion process by incorporating a secondary feature fusion structure, the PAFPN, which improves the network’s ability to localize book spines more accurately. We also introduce a cluster center initialization algorithm for the K-median clustering algorithm, allowing for the adaptive calculation of anchor box aspect ratios. This adaptive approach ensures a better alignment of the anchor boxes with the book spine dataset, thereby enhancing both model convergence speed and training effectiveness. Moreover, we expand the size of the book spine detection dataset through data augmentation techniques and employ multiple algorithms for model training and testing. The results from comparative and ablation experiments show that the proposed model achieves an mAP of 90.22% on the book spine detection dataset, surpassing other models and effectively detecting both book spines and book labels.

However, the introduction of the DCN and PAFPN has increased the model’s complexity, which could hinder practical deployment. Moving forward, we will focus on optimizing the model for lightweight applications, aiming to strike a better balance between complexity and accuracy, while facilitating deployment on embedded or mobile devices.

## Figures and Tables

**Table 1:** Hyperparameter experiments on the book spine dataset.

Variant	Batch Size	Epochs	Learning Rate	Memory (MB)	mAP (%)
1	2	48	0.005	3235	80.68
2	8	48	0.010	9128	80.36
3	16	48	0.014	17,766	79.86
4	16	96	0.014	17,766	80.20

**Table 2:** Comparison of different anchor boxes aspect ratios.

*K*	Aspect Ratio	AP (%)		mAP (%)
		Book Spine	Label	
3	{0.50, 1.00, 2.00} *	71.25	90.11	80.68
3	{1.79, 7.05, 15.94}	81.41	90.09	85.75
5	{1.48, 2.05, 4.84, 10.52, 18.00}	81.38	90.18	85.78
7	{1.33, 1.71, 2.31, 2.91, 7.44, 12.47, 18.99}	81.49	90.13	85.81

* denotes default aspect ratio without clustering.

**Table 3:** Ablation study on book spine dataset.

Methods	K-Median	DCN	PAFPN	mAP (%)	Params (MB)
1	×	×	×	80.68	41.13
2	√	×	×	85.75	41.13
3	√	√	×	90.06	41.71
4	√	×	√	90.15	44.67
5	√	√	√	90.22	45.25

**Table 4:** Comparison of oriented object detection models.

Methods	Backbone	AP (%)		mAP (%)	Params (MB)	GFLOPs	FPS
		Book Spine	Label				
R3Det	ResNet50	47.98	78.35	63.17	41.90	335.74	10.73
S2Anet	ResNet50	57.33	89.69	73.51	38.60	197.62	12.64
YOLOv8n-OBB	CSPDarknet	75.19	87.45	81.32	**10.88**	**29.63**	**26.71**
Rotated Faster R-CNN	ResNet50	64.59	89.91	77.25	41.14	198.41	12.31
RoI Transformer	ResNet50	70.85	89.45	80.15	55.13	199.52	8.14
Oriented R-CNN *	ResNet50	81.41	90.09	85.75	41.13	198.53	12.48
STD-O	ViT	89.07	88.61	88.84	30.15	120.59	3.17
Ours	ResNet50	**90.20**	**90.24**	**90.22**	45.25	195.14	10.88

* denotes the baseline model.

## Data Availability

Data are contained within the article.

## References

[1] Ji X., Zhang K., Tang L. (2023). Book spine segmentation algorithm based on improved DeepLabv3+ network. J. Comput. Appl..

[2] Cao L., Liu M., Dong Z., Yang H. Book spine recognition based on OpenCV and tesseract. Proceedings of the 2019 11th International Conference on Intelligent Human-Machine Systems and Cybernetics (IHMSC).

[3] Fang J., Du M., Pang R. (2014). Book-spine recognition by robot vision based on wavelet analysis and probability Hough transform. Comput. Eng. Sci..

[4] Cao H., Wang D., Yao Z. (2018). Auto recognition of call number for library books based on projection operator. Comput. Syst. Appl..

[5] Taira E., Uchida S., Sakoe H. Book boundary detection and title extraction for automatic bookshelf inspection. Proceedings of the 10th Korea-Japan Joint Workshop on Frontiers of Computer Vision.

[6] Chen D.M., Tsai S.S., Girod B., Hsu C.H., Kim K.H., Singh J.P. Building book inventories using smartphones. Proceedings of the International Conference on Multimedia.

[7] Cui C., Ren M. (2020). A spine location method based on text detection. Comput. Digit. Eng..

[8] Zhao X., Wang L., Zhang Y., Han X., Deveci M., Parmar M. (2024). A review of convolutional neural networks in computer vision. Artif. Intell. Rev..

[9] Chen Q., Li M., Lai Z., Zhu J., Guan L. (2024). A multi-scale target detection method using an improved faster region convolutional neural network based on enhanced backbone and optimized mechanisms. J. Imaging.

[10] Li Y. (2024). Moving object detection via feature extraction and classification. Open Comput. Sci..

[11] Wang Y., Chen X., Zhao E., Zhao C., Song M., Yu C. (2024). An unsupervised momentum contrastive learning based transformer network for hyperspectral target detection. IEEE J. Stars.

[12] Wang Y., Wang H., Zhao E., Song M., Zhao C. (2024). Tucker decomposition-based network compression for anomaly detection with large-scale hyperspectral images. IEEE J. Stars.

[13] Wang Y., Chen X., Zhao E., Song M. (2023). Self-supervised spectral-level contrastive learning for hyperspectral target detection. IEEE Trans. Geosci. Remote Sens..

[14] Yang Y., Wang Y., Wang H., Zhang L., Zhao E., Song M., Yu C. (2024). Spectral-enhanced sparse transformer network for hyperspectral super-resolution reconstruction. IEEE J. Stars.

[15] Terven J., Córdova-Esparza D.-M., Romero-González J.-A. (2023). A comprehensive review of YOLO architectures in computer vision: From YOLOv1 to YOLOv8 and YOLO-NAS. Mach. Learn. Knowl. Extr..

[16] Chen J., Chen H., Xu F., Lin M., Zhang D., Zhang L. (2024). Real-time detection of mature table grapes using ESP-YOLO network on embedded platforms. Biosyst. Eng..

[17] Situ Z., Teng S., Liao X., Chen G., Zhou Q. (2024). Real-time sewer defect detection based on YOLO network, transfer learning, and channel pruning algorithm. J. Civ. Struct. Health.

[18] Dong N., Ericsson L., Yang Y., Leonardis A., McDonagh S. (2024). Label-efficient object detection via region proposal network pre-training. Neurocomputing.

[19] Zhang Y., Lu D., Qiu X., Li F. (2023). Scattering-point-guided RPN for oriented ship detection in SAR images. Remote Sens..

[20] Zeng W., Yang Y., Zhong X. (2021). Improved Mask R-CNN based instance segmentation method for spine image of books on shelves. Appl. Res. Comput..

[21] Zeng Y., Chen Y., Yang X., Li Q., Yan J. (2024). ARS-DETR: Aspect ratio-sensitive detection transformer for aerial oriented object detection. IEEE Trans. Geosci. Remote Sens..

[22] Zeng W., Yang Y., Zhong X. (2020). A mountain-shaped network for semantic segmentation of book spines on-shelves. J. Image Signal Process..

[23] Zhou S., Sun T., Xia X., Zhang N., Huang B., Xian G., Chai X. (2022). Library on-shelf book segmentation and recognition based on deep visual features. Inform. Process. Manag..

[24] Dai J., Qi H., Xiong Y., Li Y., Zhang G., Hu H., Wei Y. Deformable convolutional networks. Proceedings of the IEEE International Conference on Computer Vision 2017.

[25] Liu S., Qi L., Qin H., Shi J., Jia J. Path aggregation network for instance segmentation. Proceedings of the IEEE Conference on Computer Vision and Pattern Recognition 2018.

[26] Wang H., Liu Z., Li Y., Zhang K. (2022). On-shelf books out-of-sequence detection based on improved YOLOv3-Tiny deep network. Mod. Electron. Tech..

[27] Xie Y., Sun T., Cui X., Deng S., Deng L., Chen B. Fast-robust book information extraction system for automated intelligence library. Proceedings of the 2021 IEEE International Conference on Artificial Intelligence and Industrial Design (AIID).

[28] Wang X., Qian S., Zhang J., Guo J. (2022). Exploration and application of library automatic book inventory checking system based on computer vision and artificial intelligence. Libr. J..

[29] Han J., Ding J., Li J., Xia G.S. (2021). Align deep features for oriented object detection. IEEE Trans. Geosci. Remote Sens..

[30] Yang X., Yan J., Feng Z., He T. R3det: Refined single-stage detector with feature refinement for rotating object. Proceedings of the AAAI Conference on Artificial Intelligence 2021.

[31] Feng S., Huang Y., Zhang N. (2024). An improved YOLOv8 obb model for ship detection through stable diffusion data augmentation. Sensors.

[32] Jiang Y., Zhu X., Wang X., Yang S., Li W., Wang H., Fu P., Luo Z. R2CNN: Rotational region cnn for arbitrarily-oriented scene text detection. Proceedings of the 2018 24th International Conference on Pattern Recognition (ICPR) 2018.

[33] Ma J., Shao W., Ye H., Wang L., Wang H., Zheng Y., Xue X. (2018). Arbitrary-oriented scene text detection via rotation proposals. IEEE Trans. Multimed..

[34] Ding J., Xue N., Long Y., Xia G.S., Lu Q. Learning RoI transformer for oriented object detection in aerial images. Proceedings of the IEEE/CVF Conference on Computer Vision and Pattern Recognition 2019.

[35] Xie X., Cheng G., Wang J., Yao X., Han J. Oriented R-CNN for object detection. Proceedings of the IEEE/CVF International Conference on Computer Vision 2021.

[36] Ultralytics. https://docs.ultralytics.com/tasks/obb/.

[37] Yang S., Pei Z., Zhou F., Wang G. Rotated faster R-CNN for oriented object detection in aerial images. Proceedings of the 2020 3rd International Conference on Robot Systems and Applications 2020.

[38] Yu H., Tian Y., Ye Q., Liu Y. Spatial transform decoupling for oriented object detection. Proceedings of the AAAI Conference on Artificial Intelligence 2024.

